# Malaria Infection, Poor Nutrition and Indoor Air Pollution Mediate Socioeconomic Differences in Adverse Pregnancy Outcomes in Cape Coast, Ghana

**DOI:** 10.1371/journal.pone.0069181

**Published:** 2013-07-22

**Authors:** Adeladza K. Amegah, Obed K. Damptey, Gideon A. Sarpong, Emmanuel Duah, David J. Vervoorn, Jouni J. K. Jaakkola

**Affiliations:** 1 Center for Environmental and Respiratory Health Research, Faculty of Medicine, University of Oulu, Oulu, Finland; 2 Department of Human Biology, School of Biological Sciences, University of Cape Coast, Cape Coast, Ghana; 3 Radel Consulting, Accra, Ghana; 4 Public Health, Institute of Health Sciences, University of Oulu, Oulu, Finland; 5 Respiratory Medicine Unit, Oulu University Hospital, Oulu, Finland; Chancellor College, University of Malawi, Malawi

## Abstract

**Background:**

The epidemiological evidence linking socioeconomic deprivation with adverse pregnancy outcomes has been conflicting mainly due to poor measurement of socioeconomic status (SES). Studies have also failed to evaluate the plausible pathways through which socioeconomic disadvantage impacts on pregnancy outcomes. We investigated the importance of maternal SES as determinant of birth weight and gestational duration in an urban area and evaluated main causal pathways for the influence of SES.

**Methods:**

A population-based cross-sectional study was conducted among 559 mothers accessing postnatal services at the four main health facilities in Cape Coast, Ghana in 2011. Information on socioeconomic characteristics of the mothers was collected in a structured questionnaire.

**Results:**

In multivariate linear regression adjusting for maternal age, parity and gender of newborn, low SES resulted in 292 g (95% CI: 440–145) reduction in birth weight. Important SES-related determinants were neighborhood poverty (221 g; 95% CI: 355–87), low education (187 g; 95% CI: 355–20), studentship during pregnancy (291 g; 95% CI: 506–76) and low income (147 g; 95% CI: 277–17). In causal pathway analysis, malaria infection (6–20%), poor nutrition (2–51%) and indoor air pollution (10–62%) mediated substantial proportions of the observed effects of socioeconomic deprivation on birth weight. Generalized linear models adjusting for confounders indicated a 218% (RR: 3.18; 95% CI: 1.41–7.21) risk increase of LBW and 83% (RR: 1.83; 95% CI: 1.31–2.56) of PTB among low income mothers. Low and middle SES was associated with 357% (RR: 4.57; 95% CI: 1.67–12.49) and 278% (RR: 3.78; 95% CI: 1.39–10.27) increased risk of LBW respectively. Malaria infection, poor nutrition and indoor air pollution respectively mediated 10–21%, 16–44% and 31–52% of the observed effects of socioeconomic disadvantage on LBW risk.

**Conclusion:**

We provide evidence of the effects of socioeconomic deprivation, substantially mediated by malaria infection, poor nutrition and indoor air pollution, on pregnancy outcomes in a developing country setting.

## Introduction

The rapid population growth of developing countries urban zones in recent years has led to the emergence and expansion of slum and squatter settlements in large parts of these areas with deplorable environmental conditions and widespread socioeconomic deprivation. Socioeconomic deprivation manifests as poor housing conditions, unemployment, low income and low educational attainment among others in these urban settlements with adverse consequences for human health. There is mounting evidence linking socioeconomic deprivation with adverse pregnancy outcomes. Factors such as area deprivation, unmarried status, low education, low income and low occupational attainment have been associated with low birth weight (birth weight <2500 grams) [1]–[7] and preterm birth (<37 weeks of gestation) [4], [7], [8]–[10] in several populations. Low birth weight (LBW) is a determinant of neonatal and infant mortality and morbidity, reduced growth, impaired immune function and poor cognitive development [11]. Preterm birth (PTB) is the leading cause of perinatal mortality and morbidity accounting for as much as 75% of perinatal deaths [12]. Preterm babies that do survive are at increased risk of neurodevelopmental impairments including cerebral palsy, mental retardation and sensory deficits, behavioural problems, and respiratory and gastrointestinal complications [13], [14]. LBW and PTB have further been associated with health problems later in childhood and adulthood.

The evidence linking socioeconomic deprivation with adverse pregnancy outcome has however been conflicting. Shavers [15], and Braveman and coworkers [16] identified the widespread application of socioeconomic status (SES) proxy measures in health disparities research without acknowledging their limitations, and interpretation of study results without specifying the particular socioeconomic factor measured as contributing to the inconsistencies in the epidemiological literature. Reviews [17], [18] summarizing the available evidence on socioeconomic disparities in adverse pregnancy outcomes shows that studies investigating this relationship typically apply a single individual-level or area-based socioeconomic variable in the measurement of SES within their study populations. Proxy measures of SES usually do not adequately reflect the social and economic standing or actual living conditions of individuals and populations under study. Blumenshine and coworkers [17] highlighted the limitations associated with use of some individual-level socioeconomic measures - occupation, income and education in capturing SES, how the measures vary in their use across populations, and the importance of these for birth outcomes. Different measures of SES capture different aspects of relative or absolute socioeconomic advantage [17] and are likely to depend on cultural and societal context. Shavers [15] asserts that choosing the best variable or approach for measuring SES is dependent in part on the relevance to the population and outcomes under study. Braveman and coworkers [16] also recommend measuring as much relevant socioeconomic information as possible in the assessment of SES influences on health outcomes. Another major shortcoming of studies exploring the relationship of SES with pregnancy outcomes is the failure to evaluate the plausible pathways through which socioeconomic disadvantage impacts on pregnancy outcomes. As noted by Kramer [18], research that identifies and quantifies the causal pathways and mechanisms whereby social disadvantage leads to higher risks of unfavorable pregnancy outcomes may eventually help to reduce current disparities and improve pregnancy outcome across the entire socioeconomic spectrum.

The objective of the present study was therefore to (a) ascertain the importance of different socioeconomic measures for fetal growth and gestational duration in Cape Coast, (b) develop a construct of SES that can strongly and consistently predict pregnancy outcomes in this and similar setting of developing countries, and (c) establish the factors mediating socioeconomic differences in pregnancy outcomes in this setting. The SES construct takes into account the population studied, the research questions asked and the outcomes of interest thereby representing a very precise and reliable measure of SES for the source population. Applying this construct and identifying the factors mediating their effects should provide a better understanding of the high burden of adverse birth outcomes in Cape Coast, and similar developing country settings whilst helping to map out appropriate intervention strategies to improve the situation.

## Methods

### Study Design and Site

A population-based cross-sectional study was conducted among mothers and newborns of the postnatal clinic of the four main health facilities in the Cape Coast metropolis - Regional Hospital, Metropolitan Hospital, University Hospital and Adisadel Urban Health Centre. The Cape Coast metropolis covers an area of 122 square kilometers with an estimated population of 169, 894 according to the 2010 census. Cape Coast is the capital of the Central Region of Ghana and the smallest of the six metropolitan areas in Ghana.

### Study Population and Data Collection

The source population comprised all nursing mothers residing in Cape Coast. Six hundred and eighty mothers who had singleton births with no gross anatomical deformities at the selected health facilities and accessing postnatal services at these same facilities were randomly sampled from a register provided by the maternity unit of the facilities. Selected mothers who visited the postnatal clinic were interviewed after been satisfied that they resided in Cape Coast and their respective neighbourhood throughout the duration of the pregnancy. The study population included five hundred and fifty nine mothers and their newborns (Overall response rate 82.2%). Of the study population, 141 (25.2%) were from the Regional Hospital, 131 (23.4%) from the Metropolitan Hospital, 139 (24.9%) from the University Hospital and 148 (26.5%) from the Adisadel Urban Health Centre. A weighting procedure based on the size of the facilities and the number of mothers who delivered at the facilities in 2010 was used to determine the number of mothers to be selected from each facility.

### Health Outcomes

The outcomes of interest were (1) fetal growth measured as birth weight (in grams) and (2) duration of pregnancy i.e. gestational age (in weeks). Information on birth weight and gestational age was retrieved from hospital records. Birth weight of the newborns was measured immediately after delivery with a regularly calibrated weighing scale which measures birth weight in kilograms. Gestational age was estimated by the health staff using the last menstrual period method.

### Determinants of Interest

The primary determinant was socioeconomic status which was operationalized as: (1) area of residence, (2) marital status, (3) education, (4) occupation and (5) income. These were assessed on the basis of information collected in a structured questionnaire which was validated in the source population. Area of residence, education and income were combined into an index of SES with four levels - low, middle, upper middle and high. The levels of SES were constructed as follows. Step one involved assigning scores to the three socioeconomic measures in the following ways: (a) for area of residence, scores of 1, 2 and 3 were assigned to poor, middle class and affluent neighborhoods respectively, (b) for income, scores of 1, 2, 3 and 4 were assigned to monthly incomes of <GH¢100 (USD$71 at the time of study), GH¢101–500 (USD$71–357), GH¢501–1000 (USD$357–714) and>GH¢1000 (USD$714 ) respectively, and (c) for education, scores of 0, 1, 2, 3 and 4 were assigned to no, primary, junior high, senior high and tertiary education respectively. Step two involved summing up the scores for each participant with the total SES scores ranging from 2 to 11. Step three involved classification of mothers with total SES scores of 2, 3 and 4 as low SES; 5, 6 and 7 as middle SES; 8 and 9 as upper middle; and 10 and 11 as high SES.

### Covariates

Covariates treated as potential confounders included maternal age, parity and gender of newborn. Covariates treated as mediating factors were reported episode of malaria during pregnancy, pre-pregnancy body mass index (BMI) and cooking fuel used during pregnancy.

### Ethics Statement

The Research Committee of the Department of Human Biology, University of Cape Coast, Cape Coast approved the study. Approval was also sought from the management of the selected facilities. Informed consent form attached to the questionnaires was used to seek the consent of all participants before inclusion in the study.

### Statistical Analysis

The relations of interest comprised maternal socioeconomic status and adverse pregnancy outcomes (reduced birth weight, LBW and PTB). We applied multivariate methods for assessing the relations of interest. First, we applied multiple linear regression to estimate the independent effects of socioeconomic characteristics on birth weight. Second, we applied generalized linear models (PROC GENMOD) with Poisson distribution and log link to estimate the independent effects of the determinants of interest on the risk of LBW and PTB. All models were adjusted for age of mother, parity and gender of infant. We performed a causal pathway analysis using the difference method [19] with computation of the percentages of mediation [20] to establish the independent and joint mediating effect of malaria episode during pregnancy, pre-pregnancy BMI, and cooking fuel used during pregnancy in the observed socioeconomic differences in the outcomes of interest. SAS version 9.3 was used to perform all the analysis.

## Results

Tables 1 and 2 present the characteristics of the study population. Majority of the mothers (59.5%) were within the age group 20–29 years. We found 39% and 19% of the mothers residing in poor and affluent neighborhoods respectively. We found 11% of the mothers having no formal education and 13% of the mothers educated up to tertiary level. Petty traders and fish mongers constituted about 41% of the mothers studied. More than half (60%) of the mothers earned a monthly income of less than GH¢100 (USD$71). A small proportion (7.5%) of the mothers earned a monthly income above GH¢1000 (USD$714). We classified 36% of the mothers as low SES. The proportion of mothers categorized as high SES was 6%. (Table 1).

**Table 1:** Characteristics of the study population.

	Total (n = 559)
Characteristic	No. (%)
**Maternal age (years)^a^**	
<20	61 (10.9)
20–29	333 (59.5)
30–39	155 (27.8)
>39	10 (1.8)
**Parity^b^**	
Primiparous	196 (35.1)
Multiparous	363 (64.9)
**Area of residence**	
Poor neighborhood	218 (39.0)
Middle class neighborhood	234 (41.9)
Affluent neighborhood	107 (19.1)
**Marital status**	
Married	406 (72.6)
Unmarried	153 (27.3)
**Education**	
None	59 (10.6)
Primary	140 (25.0)
Junior High	179 (32.0)
Senior High	109 (19.5)
Tertiary	72 (12.9)
**Occupation**	
Office worker	72 (12.9)
Hairdresser/Seamstress	123 (22.0)
Petty trader/Fish monger	227 (40.6)
Student	57 (10.2)
Housewife/Unemployed	80 (14.3)
**Income (per month)**	
<GH¢100	337 (60.3)
GH¢101–500	117 (20.9)
GH¢501–1000	63 (11.3)
> GH¢1000	42 (7.5)
**SES**	
Low	202 (36.1)
Middle	269 (48.1)
Upper middle	55 (9.8)
High	33 (5.9)

GH¢ indicates Ghana cedis.

**Table 2:** Health, nutritional and lifestyle characteristics, and cooking fuel choices of the study population.Covariates: ^a^Age group 20–29 years served as reference category; ^b^multiparous mothers served as reference category.

	Socioeconomic Status				
	Low (n = 202)	Middle (n = 269)	Upper Middle (n = 55)	High (n = 33)	Total (n = 559)
Characteristic	No. (%)	No. (%)	No. (%)	No. (%)	No. (%)
**Received prenatal care**	202 (36.1)	269 (48.1)	55 (9.8)	33 (5.9)	559 (100.0)
**Initiation of prenatal care**					
First trimester	157 (77.7)	235 (87.4)	48 (87.3)	30 (90.9)	470 (84.1)
Second trimester	44 (21.8)	34 (12.6)	7 (12.7)	3 (9.1)	88 (15.7)
Third trimester	1 (0.5)	0 (0.0)	0 (0.0)	0 (0.0)	1 (0.2)
**Malaria episode during pregnancy^a^**	107 (53.0)	138 (51.3)	19 (34.6)	6 (18.2)	270 (48.3)
**Pre-pregnancy BMI (kg/m^2^)^b^**					
Underweight (<18.5)	42 (26.8)	33 (14.0)	4 (8.3)	6 (20.0)	85 (18.1)
Normal (18.5–24.9)	45 (28.7)	88 (37.5)	17 (35.4)	3 (10.0)	153 (32.6)
Overweight (25.0–29.9)	44 (28.0)	78 (33.2)	10 (20.8)	17 (56.7)	149 (31.7)
Obese (≥30.0)	26 (16.6)	36 (15.3)	17 (35.4)	4 (13.3)	83 (17.7)
**Smoking during pregnancy**	1 (0.5)	2 (0.7)	1 (1.8)	0 (0.0)	4 (0.7)
**Cooking fuel^c^**					
LPG	9 (4.5)	48 (17.8)	22 (40.0)	20 (60.6)	99 (17.7)
Charcoal	66 (32.7)	60 (22.3)	4 (7.3)	2 (6.1)	132 (23.6)
Firewood	18 (8.9)	7 (2.6)	1 (1.8)	1 (3.0)	27 (4.8)
Charcoal & Firewood	50 (24.8)	38 (14.1)	0 (0.0)	0 (0.0)	88 (15.7)
LPG & Charcoal	48 (23.8)	103 (38.3)	28 (50.9)	10 (30.3)	189 (33.8)
LPG & Firewood	11 (5.5)	13 (4.8)	0 (0.0)	0 (0.0)	24 (4.3)

Column percentages are reported.

Covariates: ^a^Mothers reporting no episode of malaria during pregnancy served as reference category; ^b^ mothers with normal pre-pregnancy BMI served as reference category; ^c^mothers using LPG as cooking fuel served as reference category.

All the mothers reported receiving prenatal care with a high proportion (84%) initiating prenatal care early in pregnancy by attending antenatal clinic for the first time during the first trimester. Almost half (48%) of the mothers reported one or more physician-diagnosed episode of malaria during pregnancy. The proportion of mothers found to be underweight prior to being pregnant was 18%. We found 44% of the mothers using biomass fuels (charcoal, firewood or combination of both) and 18% of the mothers using liquefied petroleum gas (LPG) as cooking fuel during pregnancy. (Table 2).

Table 3 presents the characteristics of the newborns and the birth outcomes of the mothers. Over half (56%) of the newborns were boys. The proportion of infants born preterm and LBW was 41% and 17% respectively. Low and middle SES mothers delivered the highest proportion of preterm and LBW babies (86.6% and 95.8% respectively). Low SES mothers recorded the lowest mean gestational age and birth weight with high SES mothers recording the highest estimate. (Table 3).

**Table 3:** Characteristics of newborns, and pregnancy outcomes of the mothers.

	Socioeconomic Status				
	Low (n = 202)	Middle (n = 269)	Upper Middle (n = 55)	High (n = 33)	Total (n = 559)
Characteristic	No. (%)	No. (%)	No. (%)	No. (%)	No. (%)
**Gender** a					
Male					313 (56)
Female					246 (44)
**Birth order**					
First					196 (35.1)
Second & Third					291 (52.1)
Fourth & above					72 (12.9)
**Preterm birth (<37 weeks)**	91 (45.1)	109 (40.5)	18 (32.7)	13 (39.4)	231 (41.3)
**Low birth weight (<2500 grams)**	47 (23.3)	44 (16.4)	1 (1.8)	3 (9.1)	95 (17.0)
**Mean gestational age (SD)**	36.2 (3.0)	37.0 (3.1)	37.7 (2.5)	37.7 (2.5)	36.8 (3.0)
**Mean birth weight (SD)**	2923.1 (572.1)	3031.1 (596.5)	3225.7 (527.5)	3293.3 (602.1)	3026.7 (509.3)

SD indicates standard deviation.

Column percentages are reported.

a Covariates: Males served as reference category.

Tables 4 and S1 presents the effect sizes for determinants of birth weight estimated from multivariate linear regression models. Residence in poor neighborhoods resulted in a 221 g (95% CI: 355, 87) reduction in birth weight with malaria and cooking fuel mediating 13% and 15% of the observed effect respectively. Primary level educated mothers delivered babies that were 187 g (95% CI: 355, 20) smaller with pre-pregnancy BMI and cooking fuel mediating 39% and 62% of the observed effects respectively. Petty trading/fish mongering was associated with a 227 g (95% CI: 380, 74) reduction in birth weight with half of the observed effect mediated by cooking fuel. Student mothers gave birth to infants that were 291 g (95% CI: 506, 76) smaller with cooking fuel mediating 10% of the observed effect. The mediation fraction in the joint model was 14%. Housewives and unemployed mothers gave birth to babies that were 193 g (95% CI: 377, 10) smaller with pre-pregnancy BMI and cooking fuel mediating 51% and 43% of the observed effects respectively. Income of <USD$71 per month was associated with a 147 g (95% CI: 277, 17) reduction in birth weight with over half (56%) of the observed effect mediated by cooking fuel. (Table S1).

**Table 4:** Unadjusted and adjusted effect of maternal socioeconomic status (SES) on birth weight.

		Adjustment for:				
	Unadjusted	Model 1: maternal age, parity, gender of newborn	Model 2: + malaria	Model 3: + pre-pregnancy BMI	Model 4: + cooking fuel	Model 5: + malaria, pre-pregnancy BMI, cooking fuel
SES	β (95% CI)	β (95% CI)	β (95% CI)	β (95% CI)	β (95% CI)	β (95% CI)
Low	−328 (−474, −182)	−292 (−440, −145)	−258 (−405, −111)	−248 (−406, −90)	−199 (−360, −37)	−154 (−328, 20)
Middle	−220 (−360, −80)	−219 (−357, −81)	−185 (−324, −47)	−203 (−348, −57)	−162 (−306, −18)	−128 (−282, 27)
Upper middle & High	Reference	Reference	Reference	Reference	Reference	Reference

CI indicates confidence interval. GH¢ indicates Ghana cedis. SES indicates socioeconomic status.

Effect estimate (β) is in grams.

Mediation fractions (%).

Low SES: Malaria (11.6), Pre-pregnancy BMI (15.1), Cooking fuel (31.8), Joint (47.3).

Middle SES: Malaria (15.5), Pre-pregnancy BMI (7.3), Cooking fuel (26.0), Joint (41.6).

Low SES was associated with a 292 g (95% CI: 440, 145) reduction in birth weight with cooking fuel mediating 32% of the observed effect. Middle SES was also associated with a 219 g (95% CI: 357, 81) reduction in birth weight with cooking fuel mediating 26% of the observed effect. The mediation fraction in the joint models for low SES and middle SES was 47% and 42% respectively. (Table 4).

Tables 5, 6, S2 and S3 present the risk ratios for determinants of LBW and PTB calculated from generalized linear models. Education up to primary level was associated with a 95% (RR = 1.95, 95% CI: 0.97, 3.94) increased risk of LBW. Work as a petty trader or fish monger was associated with a 230% (RR = 3.30, 95% CI: 1.22, 8.89) increased risk of LBW with over half (52%) of the observed effect mediated by cooking fuel. Homemaking and unemployment was associated with a 225% (RR = 3.25, 95% CI: 1.15, 9.14) increased risk of LBW with pre-pregnancy BMI and cooking fuel mediating 44% and 39% of the observed effect respectively. Studentship during pregnancy was associated with a 166% (RR = 2.66, 95% CI: 0.92, 7.71) increased risk of LBW. Income of <USD$71 per month was associated with a 218% (RR = 3.18, 95% CI: 1.41, 7.21) increased risk of LBW with 42% of the observed effect mediated by cooking fuel. The mediation fraction in the joint model was more than half (60%). (Table S2).

**Table 5:** Unadjusted and adjusted risk of low birth weight (LBW) attributable to maternal socioeconomic status (SES).

		Adjustment for:				
	Unadjusted	Model 1: maternal age, parity, gender of newborn	Model 2: + malaria	Model 3: + pre-pregnancy BMI	Model 4: + cooking fuel	Model 5: + malaria, pre-pregnancy BMI, cooking fuel
SES	RR (95% CI)	RR (95% CI)	RR (95% CI)	RR (95% CI)	RR (95% CI)	RR (95% CI)
Low	4.90 (1.82, 13.17)	4.57 (1.67, 12.49)	3.83 (1.42, 10.34)	3.64 (1.33, 10.01)	3.09 (1.12, 8.55)	2.29 (0.81, 6.48)
Middle	3.58 (1.33, 9.67)	3.78 (1.39, 10.27)	3.28 (1.23, 8.79)	3.02 (1.11, 8.26)	2.91 (1.06, 7.97)	2.09 (0.75, 5.78)
Upper middle & High	1.00	1.00	1.00	1.00	1.00	1.00

CI indicates confidence interval. GH¢ indicates Ghana cedis. RR indicates risk ratio. SES indicates socioeconomic status.

Mediation fractions (%).

Low SES: Malaria (20.7), Pre-pregnancy BMI (26.1), Cooking fuel (41.5), Joint (63.9).

Middle SES: Malaria (18.0), Pre-pregnancy BMI (27.3), Cooking fuel (31.3), Joint (60.8).

**Table 6:** Unadjusted and adjusted risk of preterm birth (PTB) attributable to maternal socioeconomic status (SES).

		Adjustment for:				
	Unadjusted	Model 1: maternal age, parity	Model 2: + malaria	Model 3: + pre-pregnancy BMI	Model 4: + cooking fuel	Model 5: + malaria, pre-pregnancy BMI, cooking fuel
SES	RR (95% CI)	RR (95% CI)	RR (95% CI)	RR (95% CI)	RR (95% CI)	RR (95% CI)
Low	1.23 (0.90, 1.69)	1.26 (0.91, 1.73)	1.26 (0.91, 1.74)	1.16 (0.84, 1.62)	1.05 (0.73, 1.51)	1.06 (0.74, 1.51)
Middle	1.15 (0.84, 1.57)	1.16 (0.85, 1.59)	1.16 (0.85, 1.60)	1.24 (0.91, 1.69)	1.07 (0.76, 1.49)	1.20 (0.86, 1.67)
Upper middle & High	1.00	1.00	1.00	1.00	1.00	1.00

CI indicates confidence interval. GH¢ indicates Ghana cedis. RR indicates risk ratio. SES indicates socioeconomic status.

Low and middle SES was respectively associated with a 357% (RR = 4.57, 95% CI: 1.67, 12.49) and 278% (RR = 3.78, 95% CI: 1.39, 10.27) increased risk of LBW with cooking fuel mediating 42% and 31% of the observed effects in both relationships respectively. The mediation fraction in the joint models for both low SES and middle SES was more than half (64% and 61% respectively). (Table 5).

Monthly income of <USD$71 was associated with 83% (RR = 1.83; 95% CI: 1.31, 2.56) increased risk of PTB with 17% and 22% of the observed effect mediated by pre-pregnancy BMI and cooking fuel respectively. The mediation fraction estimated from the joint model was 30%. (Table S3).

Low and middle SES was associated with a 26% (RR = 1.26, 95% CI: 0.91, 1.73) and 16% (RR = 1.16, 95% CI: 0.85, 1.59) increased risk of PTB respectively; albeit not statistically significant. (Table 6).

## Discussion

Our population-based study of urban Ghanaian women and their newborns provides evidence of the importance of maternal socioeconomic characteristics for pregnancy outcomes. Area of residence, education, occupation and income were independent determinants of birth weight and risk of LBW in this population. The construct of SES was also a strong determinant of birth weight and LBW risk with low and middle SES respectively associated with 357% (RR = 4.57, 95% CI: 1.67, 12.49) and 278% (RR = 3.78, 95% CI: 1.39, 10.27) increased risk of LBW. Income was the only socioeconomic characteristic associated with statistically significant increased risk of PTB in this population. Malaria infection, poor pre-pregnancy nutrition and indoor air pollution substantially mediated observed effects of socioeconomic disadvantage on fetal growth and gestational duration in this population.

### Validity of Results

The study represents a defined catchment area because consecutive mothers giving birth at the main health facilities in the study area were selected. A weighting procedure based on the size of the facilities and the number of mothers delivering at these facilities was applied to determine a representative sample from each of the study sites. The population-based nature of the study together with the high response rate (82.2%) minimizes selection bias. Even though our study was conducted in an urban area it is still likely that there are some mothers who have barriers preventing them from accessing postnatal services such as lack of money for transport to the health facility. Unfortunately we could not track these mothers for inclusion in our study because the delivery registers at the health facilities do not contain information on the exact residential address of the mothers registered. The potential for selection bias due to the exclusion of mothers not seeking postnatal care from the study is nonetheless minimized in our study. This is due to the high patronage of postnatal services in Cape Coast and other urban settings of Ghana as a result of the high level of awareness of Reproductive and Child Health (RCH) service provision and benefits among urban mothers and the ease of access to RCH services. Many mothers in several urban areas of Ghana do not have to travel long distances to access RCH services due to the wide distribution of health services as a whole in most urban areas. Most mothers are even able to walk to these RCH service provision centers without the need for transport. Finally, mothers giving birth at home and possibly not accessing postnatal care should not be a major concern in our study because unlike rural areas of Ghana, very few mothers deliver at home in urban areas. This small number of home deliveries in urban areas of Ghana usually occurs by accident with most mothers immediately checking in at the nearest health facility due to the ease of access to health facilities.

The outcomes were measured and recorded independently from the study with negligible measurement error in all the study sites and thus represent an objective variable. There are limitations associated with the use of the last menstrual period method for estimating gestational age and can obviously result in invalid estimates of gestation but as documented by Lynch and Zhang [21] so do other methods such as ultrasound measurements. Lynch and Zhang [21] suggest that some findings are an artefact of the choice of method used in estimating gestational age rather than evidence of causality. We do not however think the potential bias associated with the method applied solely explains our findings.

The SES construct combines data on the mothers area of residence; an area-based measure, and their educational attainment and income levels; individual-level measures, and is a context-specific, culturally sensitive objective variable that captures all aspects of social and economic standing in this population. Occupational class which has traditionally been used in constructing SES indices in various settings was not factored in our construct. With the exception of office work, all the other occupational groups are low income generating activities thereby ranking mothers in any of these jobs on a similar scale in terms of relative social position, economic success and actual standard of living. Our findings on the association of the various occupational groups with statistically significant reductions in birth weight in a way confirm this assertion. In such situations therefore, income is an adequate measure as it captures some of the very aspects of SES that occupational class will capture in any construct whilst also accounting for any small differences in the occupational groups with respect to these aspects of SES. We also did not factor marital status into the construct because our lack of data on paternal socioeconomic characteristics meant we were unable to determine how marital status of mothers translates into for example improved household income, better standard of living and social recognition without a complement of their spouses’ data.

We applied multivariate methods to adjust for the potential confounding effect of maternal age, parity and gender of neonate. We did not control for smoking during pregnancy because only 0.7% of the mothers smoked during pregnancy. We employed the difference approach which was first proposed by Judd and Kenny [19] and is commonly used in epidemiologic research to establish the mediating effects of malaria infection during pregnancy, pre-pregnancy nutritional status, and indoor air pollution exposure assessed by cooking fuel used during pregnancy. We computed and reported the percentages of mediation which gives a measure of the proportion of the overall effect mediated by the variable of interest in the causal pathway.

### Synthesis with Previous Knowledge

Our study shows how socioeconomic deprivation adversely affects fetal development. Area of residence, education, occupation and income were determinants of birth weight and gestational length in this population. Residing in poor neighborhoods was associated with a mean 221 g reduction in birth weight. Previous studies have also found women living in deprived areas delivering the majority of LBW infants [5], [6], [8]. The Cape Coast area as a whole is a deprived urban settlement with very limited affluent neighborhoods. Deprived zones of Ghanaian urban settlements including Cape Coast are noted for deplorable social and environmental conditions with attendant poor indoor and outdoor air quality, high malaria transmission levels and repeated infections. Poor nutritional practices and associated nutritional deficits due to widespread poverty and ignorance are also common in these areas. All these factors are proposed mediators through which neighborhood deprivation can impact on birth outcomes and could explain our findings. In the present study, malaria infection during pregnancy, poor pre-pregnancy nutritional status and indoor air pollution independently explained about 13%, 5% and 15% of the observed effect respectively. Jointly they mediated more than a quarter (29%) of the observed effect.

Low income was associated with increased risk of LBW and PTB in this population. The adverse perinatal effects of low income levels of mothers and households have been noted in diverse populations; nationwide in the US [4], London, UK [9], Dhaka, Bangladesh [1], [2], and in Hatyai city, Thailand [22]. Low income translates into poor housing, material deprivation, patronage of biomass fuel, poor nutrition, limited access to prenatal care, low social support and psychosocial stress. These factors have been shown to adversely affect reproductive health and birth outcomes. In our study, poor pre-pregnancy nutrition for instance mediated about 28% and 17% of the observed effect of low income on risk of LBW and PTB respectively. Use of biomass fuels for cooking explained about 42% and 22% of the effect of low income on the risk of LBW and PTB respectively.

Petty traders/fishmongers and housewives/unemployed mothers were at particular risk of delivering smaller babies. Petty trading and fish mongering are low income generating activity in the study area. Homemaking and unemployment especially among mothers whose spouses are not in gainful employment obviously translates into poor family income. The adverse perinatal effects of low income has been shown in the present study and previous reviewed, and is not surprising that these group of mothers were vulnerable. The low income levels of these mothers also translates into poor nutritional practices and patronage of biomass fuels which the present study found to substantially mediate the observed effects of low occupational attainment on adverse pregnancy outcomes.

Student mothers were also at risk of delivering smaller babies and were a vulnerable group possibly due to the stressful experience of combining studies with pregnancy. Stress in pregnancy is well documented to adversely affect pregnancy outcomes. Also, it is not unusual to find resident student mothers who are married to be anxious about the welfare of their spouse and children back home which can further exacerbates stress levels. Malaria infections, poor pre-pregnancy nutrition and indoor air pollution jointly explained only about 14% of the observed effect in this group of mothers and certainly strengthens our stress hypothesis. Our finding on the association of studentship during pregnancy with reduced birth weight is to the best of our knowledge the first of such report in the epidemiological literature. This certainly underscores the need to further explore the relationship especially in areas where women are not presented with the opportunity to take leave from studies when pregnant to generate the evidence base for public health action to safeguard maternal and perinatal health.

In applying our construct of maternal SES, we found low and middle SES to be associated with statistically significant reductions in birth weight and increased risk of LBW in this population. Malaria infection, poor pre-pregnancy nutrition and indoor air pollution jointly explained between 42% and 64% of the observed effects in the models. Indoor air pollution assessed by biomass fuel use during pregnancy however appears to be the most important mediating factor explaining between 26% and 42% of the observed associations in the models. Studies in India [23], [24], Nigeria [25] and Mexico [26] using different proxies and constructs of SES have also associated low SES with reduced birth weight and risk of LBW. A study in Accra, Ghana found use of biomass fuel, and cooking area particulate matter levels to be high in two low SES neighborhoods of the city [27]. Use of charcoal for cooking, and garbage burning at home have also been shown in Accra to adversely affect birth weight with the authors identifying low social class mothers as the group patronizing biomass fuels mostly [28]. Collecting as much relevant socioeconomic information as possible and summarizing them to develop an SES construct for the source population, and establishing the main causal pathways in our setting certainly strengthens the study findings and the epidemiological evidence greatly.

In conclusion, our study provides evidence that socioeconomic deprivation is a strong determinant of adverse pregnancy outcomes in this Ghanaian setting. We also show that malaria infection, poor pre-pregnancy nutrition and indoor air pollution which have been associated with adverse birth outcomes in several settings are important pathways through which socioeconomic disadvantage impact on fetal growth and gestational duration in our setting. Improving social status and income levels of women, and addressing household and neighborhood poverty are thus important to curbing the high burden of adverse birth outcomes in Cape Coast and similar deprived zones of developing countries.

## Supporting Information

Table S1 Unadjusted and adjusted effect of maternal socioeconomic characteristics on birth weight.
(DOCX)

Table S2 Unadjusted and adjusted risk of low birth weight (LBW) attributable to maternal socioeconomic characteristics.
(DOCX)

Table S3 Unadjusted and adjusted risk of preterm birth (PTB) attributable to maternal socioeconomic characteristics.
(DOCX)

## References

[1] Khatun S, Rahman M (2008) Socio-economic determinants of low birth weight in Bangladesh: A multivariate approach. Bangladesh Med Res Counc Bull 34: 81–86. 19476252 10.3329/bmrcb.v34i3.1857

[2] Karim E, Mascie-Taylor CG (1997) The association between birth weight, socio-demographic variables and maternal anthropometry in an urban sample from Dhaka, Bangladesh. Ann Hum Biol 24: 387–401. 9300116 10.1080/03014469700005152

[3] Nordström ML, Cnattingius S (1996) Effects on birthweights of maternal education, socio-economic status, and work-related characteristics. Scand J Soc Med 24: 55–61. 8740877 10.1177/140349489602400109

[4] Parker JD, Schoendorf KC, Kiely JL (1994) Associations between measures of socioeconomic status and low birth weight, small for gestational age, and premature delivery in the United States. Ann Epidemiol 4: 271–278. 7921316 10.1016/1047-2797(94)90082-5

[5] Pattenden S, Dolk H, Vrijheid M (1999) Inequalities in low birth weight: parental social class, area deprivation, and “lone mother” status. J Epidemiol Community Health 53: 355–358. 10396482 10.1136/jech.53.6.355 PMC1756885

[6] Dibben CH, Sigala M, Macfarlane A (2006) Area deprivation, individual factors and LBW in England: is there evidence of an “area effect”? J Epidemiol Community Health 60: 1053–1059. 17108301 10.1136/jech.2005.042853 PMC2465519

[7] Koupilova I, Rahu K, Rahu M, Karroc H, Leon DA (2000) Social determinants of birthweight and length of gestation in Estonia during the transition to democracy. Int J Epidemiol 29: 118–124. 10750613 10.1093/ije/29.1.118

[8] Thompson JM, Irgens LM, Rasmussen S, Daltveit AK (2006) Secular trends in socio-economic status and the implications for preterm birth. Paediatr Perinat Epidemiol 20: 182–187. 16629692 10.1111/j.1365-3016.2006.00711.x

[9] Peacock JL, Bland JM, Anderson HR (1995) Preterm delivery: effects of socioeconomic factors, psychological stress, smoking, alcohol, and caffeine. BMJ 311: 531–535. 7663207 10.1136/bmj.311.7004.531 PMC2550601

[10] Olsen P, Laara E, Rantakallio P, Järvelin MR, Sarpola A, et al (1995) Epidemiology of preterm delivery in two birth cohorts with an interval of 20 years. Am J Epidemiol 142: 1184–1193. 7485065 10.1093/oxfordjournals.aje.a117577

[11] ACC/SCN (United Nations Administrative Committee on Coordination Sub-committee on Nutrition) (2000) Low birth weight: Report of a Meeting in Dhaka, Bangladesh on 14–17 June 1999. ACC/SCN Nutrition Policy Paper 18. Edited by Pojda J, Kelley L. Geneva: ACC/SCN in collaboration with ICDDR,B. Available: http://www.unscn.org/layout/modules/resources/files/Policy_paper_No_18.pdf. Accessed 20 October 2012.

[12] Ananth CV, Vintzileos AM (2006) Epidemiology of preterm birth and its clinical subtypes. J Matern Fetal Neonatal Med 19: 773–782. 17190687 10.1080/14767050600965882

[13] Goldenberg RL, Culhane JF, Iams JD, Romero R (2008) Epidemiology and causes of preterm birth. Lancet 371: 75–84. 18177778 10.1016/S0140-6736(08)60074-4 PMC7134569

[14] Saigal S, Doyle LW (2008) An overview of mortality and sequelae of preterm birth from infancy to adulthood. Lancet 371: 261–269. 18207020 10.1016/S0140-6736(08)60136-1

[15] Shavers VL (2007) Measurement of socioeconomic status in health disparities research. J Natl Med Assoc 99: 1013–1023. 17913111 PMC2575866

[16] Braveman PA, Cubbin C, Egerter S, Chideya S, Marchi KS, et al (2005) Socioeconomic status in health research: one size does not fıt all. JAMA 294: 2879–2888. 16352796 10.1001/jama.294.22.2879

[17] Blumenshine P, Egerter S, Barclay CJ, Cubbin C, Braveman PA (2010) Socioeconomic disparities in adverse birth outcomes: a systematic review. Am J Prev Med 39: 263–272. 20709259 10.1016/j.amepre.2010.05.012

[18] Kramer MS, Seguin L, Lydon J, Goulet L (2000) Socioeconomic disparities in pregnancy outcome: why do the poor fare so poorly? Paediatr Perinat Epidemiol 14: 194–210. 10949211 10.1046/j.1365-3016.2000.00266.x

[19] Judd CM, Kenny DA (1981) Process Analysis: Estimating mediation in treatment evaluations. Eval Rev 5: 602–619.

[20] van de Mheen H, Stronks J, van den Bos J, Mackenbach JP (1997) The contribution of childhood environment to the explanation of socioeconomic inequalities in health in adult life: a retrospective study. Soc Sci Med 44: 13–24.

[21] Lynch CD, Zhang J (2007) The research implications of the selection of a gestational age estimation method. Paediatr Perinat Epidemiol 21: 86–96. 17803622 10.1111/j.1365-3016.2007.00865.x

[22] Tuntiseranee P, Olsen J, Chongsuvivatwong V, Limbutara S (1999) Socioeconomic and work related determinants of pregnancy outcome in southern Thailand. J Epidemiol Community Health 53: 624–629. 10616674 10.1136/jech.53.10.624 PMC1756780

[23] Deshmukh JS, Motghare DD, Zodpey SP, Wadhva SK (1998) Low birth weight and associated maternal factors in an urban area. Indian Pediatr 35: 33–36. 9707902

[24] Mumbare SS, Maindarkar G, Darade R, Yenge S, Tolani MK, et al (2012) Maternal risk factors associated with term low birth weight neonates: a matched-pair case control study. Indian Pediatr 49: 25–28. 21719926 10.1007/s13312-012-0010-z

[25] Olusanya BO, Ofovwe GE (2010) Predictors of preterm births and low birthweight in an inner-city hospital in sub-Saharan Africa. Matern Child Health J 14: 978–986. 19795198 10.1007/s10995-009-0528-4

[26] Torres-Arreola LP, Constantino-Casas P, Flores-Hernández S, Villa-Barragán JP, Rendón-Macías E (2005) Socioeconomic factors and low birth weight in Mexico. BMC Public Health 5: 20. Available: http://www.biomedcentral.com/1471-2458/5/20. Accessed 20 October 2012. 10.1186/1471-2458-5-20 PMC554974 15745443

[27] Zhou Z, Dionisio KL, Arku RE, Quaye A, Hughes FA, et al (2011) Household and community poverty, biomass use, and air pollution in Accra, Ghana. Proc Natl Acad Sci USA 108: 11028–11033. 21690396 10.1073/pnas.1019183108 PMC3131368

[28] Amegah AK, Jaakkola JJ, Quansah R, Norgbe GK, Dzodzomenyo M (2012) Cooking fuel choices and garbage burning practices as determinants of birth weight: a cross-sectional study in Accra, Ghana. Environ Health 11: 78. Available: http://www.ehjournal.net/content/11/1/78. Accessed 20 October 2012. 10.1186/1476-069X-11-78 PMC3533864 23075225

