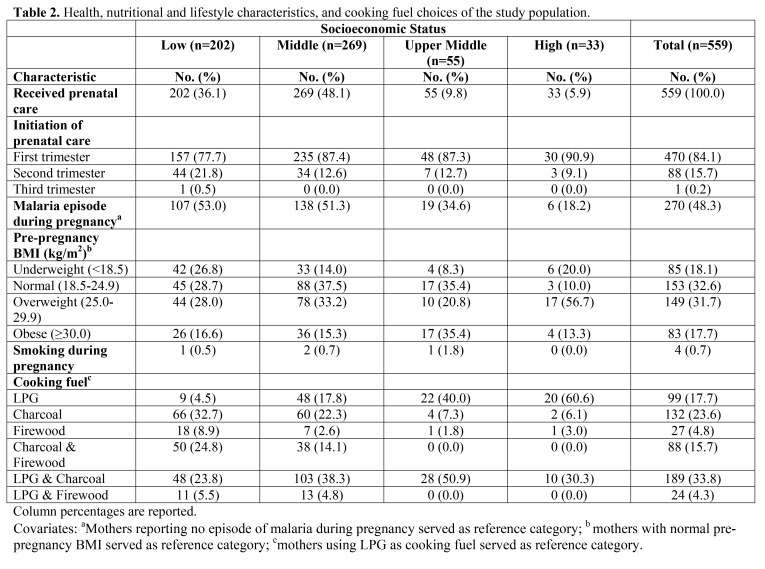# Correction: Malaria Infection, Poor Nutrition and Indoor Air Pollution Mediate Socioeconomic Differences in Adverse Pregnancy Outcomes in Cape Coast, Ghana

**DOI:** 10.1371/annotation/5e96cde8-0fc2-4bba-a8a9-e2157baab074

**Published:** 2013-12-30

**Authors:** Adeladza K. Amegah, Obed K. Damptey, Gideon A. Sarpong, Emmanuel Duah, David J. Vervoorn, Jouni J. K. Jaakkola

Text from the footnote of Table 1 was erroneously included in the title of Table 2. Please see the corrected tables here: 

Table 1: 

**Figure:**
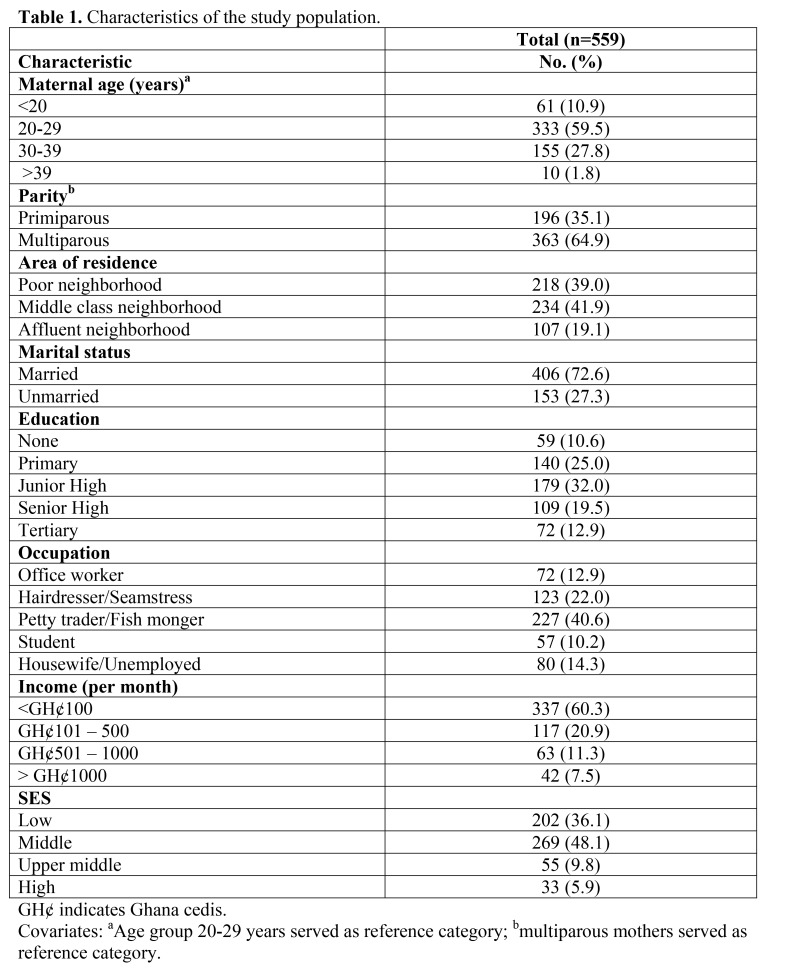


Table 2: 

**Figure:**