# Supplementary material for: Malaria Infection, Poor Nutrition and Indoor Air Pollution Mediate Socioeconomic Differences in Adverse Pregnancy Outcomes in Cape Coast, Ghana
Source: PLoS One. 2013 Jul 22;8(7):e69181. doi: 10.1371/journal.pone.0069181 (PMC3718681; doi:10.1371/journal.pone.0069181)
Supplement: Table S1 — Unadjusted and adjusted effect of maternal socioeconomic characteristics on birth weight. (DOCX) [file pone.0069181.s001.docx]

**Table S1.** Unadjusted and adjusted effect of maternal socioeconomic characteristics on birth weight.

|  |  | **Adjustment for:** | | | | |
| --- | --- | --- | --- | --- | --- | --- |
|  | **Unadjusted** | **Model 1:** maternal age, parity, gender of newborn | **Model 2:** + malaria | **Model 3:** + pre-pregnancy BMI | **Model 4:** + cooking fuel | **Model 5:** +malaria, pre-pregnancy BMI, cooking fuel |
| **Characteristic** | **β (95% CI)** | **β (95% CI)** | **β (95% CI)** | **β (95% CI)** | **β (95% CI)** | **β (95% CI)** |
| **Area of residence** |  |  |  |  |  |  |
| Poor | -242 (-377, -107) | -221 (-355, -87) | -193 (-327, -60) | -209 (-353, -66) | -187 (-321, -54) | -156 (-302, -9) |
| Middle class | -51 (-184, 83) | -45 (-175, 86) | -57 (-186, 73) | -72 (-211, 68) | -31 (-161, 98) | -61 (-200, 77) |
| Affluent | Reference | Reference | Reference | Reference | Reference | Reference |
| **Marital status** |  |  |  |  |  |  |
| Married | Reference | Reference | Reference | Reference | Reference | Reference |
| Unmarried | -216 (-325, -108) | -82 (-215, 50) | -70 (-201, 61) | -90 (-237, 57) | -38 (-171, 95) | -43 (-191, 106) |
| **Education** |  |  |  |  |  |  |
| Tertiary | Reference | Reference | Reference | Reference | Reference | Reference |
| None | -66 (-267, 136) | -77 (-279, 124) | -50 (-249, 150) | -69 (-285, 147) | 52 (-163, 267) | 51 (-180, 281) |
| Primary | -215 (-382, -49) | -187 (-355, -20) | -150 (-316, 17) | -115 (-291, 60) | -71 (-252, 110) | -4 (-193, 186) |
| Junior High | -90 (-250, 70) | -82 (-241, 78) | -48 (-207, 111) | -20 (-186, 146) | 16 (-153, 186) | 81 (-97, 258) |
| Senior High | 67 (-108, 241) | 73 (-100, 245) | 106 (-66, 278) | 131 (-48, 310) | 124 (-51, 299) | 192 (11, 373) |
| **Occupation** |  |  |  |  |  |  |
| Office worker | Reference | Reference | Reference | Reference | Reference | Reference |
| Hairdresser/Seamstress | -253 (-423, -83) | -233 (-400, -66) | -203 (-369, -37) | -182 (-357, -6) | -151 (-323, 21) | -96 (-277, 86) |
| Petty trader/Fish monger | -207 (-362, -52) | -227 (-380, -74) | -193 (-345, -40) | -186 (-345, -28) | -113 (-278, 51) | -76 (-248, 96) |
| Student | -422 (-626, -219) | -291 (-506, -76) | -274 (-487, -61) | -286 (-517, -55) | -261 (-475, -48) | -249 (-478, -20) |
| Housewife/Unemployed | -220 (-407, -34) | -193 (-377, -10) | -162 (-345, 21) | -95 (-293, 103) | -111 (-300, 77) | -12 (-214, 190) |
| **Income** |  |  |  |  |  |  |
| <GH¢100 | -212 (-340, -83) | -147 (-277, -17) | -131 (-259, -2) | -87 (-225, 50) | -65 (-202, 73) | -19 (-163, 125) |
| GH¢100-500 | -96 (-250, 59) | -102 (-253, 50) | -96 (-246, 53) | -69 (-227, 90) | -24 (-181, 132) | -10 (-173, 152) |
| > GH¢500 | Reference | Reference | Reference | Reference | Reference | Reference |

CI indicates confidence interval. GH¢ indicates Ghana cedis.

Effect estimate (β) is in grams.

Mediation fractions (%).

Residence in poor neighborhood: Malaria (12.7), Pre-pregnancy BMI (5.4), Cooking fuel (15.4), Joint (29.4).

Primary education: Malaria (19.8), Pre-pregnancy BMI (38.5), Cooking fuel (62.0), Joint (97.9).

Work as hairdresser/seamstress: Malaria (12.9), Pre-pregnancy BMI (21.9), Cooking fuel (35.2), Joint (58.8).

Work as petty trader/fish monger: Malaria (15.0), Pre-pregnancy BMI (18.1), Cooking fuel (50.2), Joint (66.5).

Homemaking/ unemployment: Malaria (16.1), Pre-Pregnancy BMI (50.8), Cooking fuel (42.5), Joint (93.8).

Studentship: Malaria (5.8), Pre-pregnancy BMI (1.7), Cooking fuel (10.3), Joint (14.4).

Income of <GH¢100: Malaria (10.9), Pre-pregnancy BMI (40.8), Cooking fuel (55.8), Joint (87.1).
