# Supplementary material for: Malaria Infection, Poor Nutrition and Indoor Air Pollution Mediate Socioeconomic Differences in Adverse Pregnancy Outcomes in Cape Coast, Ghana
Source: PLoS One. 2013 Jul 22;8(7):e69181. doi: 10.1371/journal.pone.0069181 (PMC3718681; doi:10.1371/journal.pone.0069181)
Supplement: Table S2 — Unadjusted and adjusted risk of low birth weight (LBW) attributable to maternal socioeconomic characteristics. (DOCX) [file pone.0069181.s002.docx]

**Table S2.** Unadjusted and adjusted risk of low birth weight (LBW) attributable to maternal socioeconomic characteristics.

|  |  | **Adjustment for:** | | | | |
| --- | --- | --- | --- | --- | --- | --- |
|  | **Unadjusted** | **Model 1:** maternal age, parity, gender of newborn | **Model 2:** + malaria | **Model 3:** + pre-pregnancy BMI | **Model 4:** + cooking fuel | **Model 5:** +malaria, pre-pregnancy BMI, cooking fuel |
| **Characteristic** | **RR (95% CI)** | **RR (95% CI)** | **RR (95% CI)** | **RR (95% CI)** | **RR (95% CI)** | **RR (95% CI)** |
| **Area of residence** |  |  |  |  |  |  |
| Poor | 1.57 (0.91, 2.71) | 1.51 (0.89, 2.55) | 1.32 (0.78, 2.21) | 1.44 (0.77, 2.70) | 1.34 (0.80, 2.26) | 1.10 (0.58, 2.09) |
| Middle class | 1.09 (0.61, 1.94) | 1.07 (0.62, 1.85) | 1.18 (0.70, 1.99) | 1.02 (0.54, 1.93) | 1.01 (0.59, 1.74) | 0.99 (0.53, 1.82) |
| Affluent | 1.00 | 1.00 | 1.00 | 1.00 | 1.00 | 1.00 |
| **Marital status** |  |  |  |  |  |  |
| Married | 1.00 | 1.00 | 1.00 | 1.00 | 1.00 | 1.00 |
| Unmarried | 1.98 (1.38, 2.83) | 1.16 (0.72, 1.87) | 1.07 (0.66, 1.73) | 1.13 (0.62, 2.03) | 1.00 (0.63, 1.57) | 0.93 (0.52, 1.64) |
| **Education** |  |  |  |  |  |  |
| Tertiary | 1.00 | 1.00 | 1.00 | 1.00 | 1.00 | 1.00 |
| None | 1.19 (0.51, 2.80) | 1.41 (0.60, 3.35) | 1.21 (0.53, 2.78) | 1.12 (0.43, 2.90) | 0.84 (0.35, 2.01) | 0.56 (0.22, 1.46) |
| Primary | 2.17 (1.11, 4.21) | 1.95 (0.97, 3.94) | 1.66 (0.83, 3.32) | 1.57 (0.75, 3.26) | 1.18 (0.57, 2.41) | 0.88 (0.42, 1.87) |
| Junior High | 1.30 (0.65, 2.60) | 1.41 (0.69, 2.87) | 1.23 (0.61, 2.46) | 1.13 (0.54, 2.35) | 0.90 (0.44, 1.85) | 0.64 (0.30, 1.35) |
| Senior High | 0.68 (0.29, 1.63) | 0.74 (0.30, 1.82) | 0.66 (0.28, 1.58) | 0.60 (0.24, 1.54) | 0.60 (0.24, 1.46) | 0.44 (0.18, 1.09) |
| **Occupation** |  |  |  |  |  |  |
| Office worker | 1.00 | 1.00 | 1.00 | 1.00 | 1.00 | 1.00 |
| Hairdresser/Seamstress | 2.41 (0.85, 6.88) | 2.46 (0.87, 6.94) | 2.07 (0.75, 5.74) | 1.89 (0.65, 5.52) | 1.79 (0.63, 5.05) | 1.24 (0.44, 3.54) |
| Petty trader/Fish monger | 3.05 (1.13, 8.20) | 3.30 (1.22, 8.89) | 2.89 (1.09, 7.65) | 2.93 (1.08, 7.97) | 2.10 (0.77, 5.69) | 1.78 (0.66, 4.83) |
| Student | 4.79 (1.70, 13.49) | 2.66 (0.92, 7.71) | 2.52 (0.89, 7.13) | 2.44 (0.78, 7.63) | 2.30 (0.78, 6.75) | 2.13 (0.70, 6.51) |
| Housewife/Unemployed | 3.97 (1.41, 11.14) | 3.25 (1.15, 9.14) | 3.03 (1.10, 8.32) | 2.27 (0.76, 6.78) | 2.38 (0.85, 6.67) | 1.68 (0.57, 4.92) |
| **Income** |  |  |  |  |  |  |
| <GH¢100 | 3.81 (1.71, 8.48) | 3.18 (1.41, 7.21) | 2.93 (1.31, 6.55) | 2.58 (1.14, 5.85) | 2.26 (0.96, 5.34) | 1.88 (0.81, 4.39) |
| GH¢100-500 | 2.21 (0.89, 5.48) | 2.39 (0.96, 5.94) | 2.33 (0.95, 5.71) | 2.06 (0.82, 5.19) | 1.81 (0.73, 4.50) | 1.63 (0.66, 4.03) |
| > GH¢500 | 1.00 | 1.00 | 1.00 | 1.00 | 1.00 | 1.00 |

CI indicates confidence interval. GH¢ indicates Ghana cedis. RR indicates risk ratio.

Mediation fractions (%).

Work as petty trader/fish monger: Malaria (17.8), Pre-pregnancy BMI (16.1), Cooking fuel (52.2), Joint (66.1).

Homemaking/unemployment: Malaria (9.8), Pre-pregnancy BMI (43.6), Cooking fuel (38.7), Joint (69.8).

Income of <GH¢100: Malaria (11.5), Pre-pregnancy BMI (27.5), Cooking fuel (42.2), Joint (59.6).
