# Supplementary material for: Malaria Infection, Poor Nutrition and Indoor Air Pollution Mediate Socioeconomic Differences in Adverse Pregnancy Outcomes in Cape Coast, Ghana
Source: PLoS One. 2013 Jul 22;8(7):e69181. doi: 10.1371/journal.pone.0069181 (PMC3718681; doi:10.1371/journal.pone.0069181)
Supplement: Table S3 — Unadjusted and adjusted risk of preterm birth (PTB) attributable to maternal socioeconomic characteristics. (DOCX) [file pone.0069181.s003.docx]

**Table S3.** Unadjusted and adjusted risk of preterm birth (PTB) attributable to maternal socioeconomic characteristics.

|  |  | **Adjustment for:** | | | | |
| --- | --- | --- | --- | --- | --- | --- |
|  | **Unadjusted** | **Model 1:** maternal age, parity | **Model 2:** + malaria | **Model 3:** + pre-pregnancy BMI | **Model 4:** + cooking fuel | **Model 5:** +malaria, pre-pregnancy BMI, cooking fuel |
| **Characteristic** | **RR (95% CI)** | **RR (95% CI)** | **RR (95% CI)** | **RR (95% CI)** | **RR (95% CI)** | **RR (95% CI)** |
| **Area of residence** |  |  |  |  |  |  |
| Poor | 0.81 (0.62, 1.06) | 0.79 (0.60, 1.03) | 0.78 (0.60, 1.03) | 0.86 (0.66, 1.12) | 0.76 (0.58, 1.00) | 0.85 (0.64, 1.12) |
| Middle class | 0.98 (0.76, 1.26) | 0.95 (0.74, 1.22) | 0.95 (0.74, 1.23) | 1.05 (0.82, 1.33) | 0.93 (0.73, 1.19) | 1.03 (0.81, 1.31) |
| Affluent | 1.00 | 1.00 | 1.00 | 1.00 | 1.00 | 1.00 |
| **Marital status** |  |  |  |  |  |  |
| Married | 1.00 | 1.00 | 1.00 | 1.00 | 1.00 | 1.00 |
| Unmarried | 0.94 (0.75, 1.17) | 0.88 (0.68, 1.14) | 0.88 (0.68, 1.14) | 0.98 (0.74, 1.30) | 0.83 (0.63, 1.07) | 0.93 (0.70, 1.25) |
| **Education** |  |  |  |  |  |  |
| Tertiary | 1.00 | 1.00 | 1.00 | 1.00 | 1.00 | 1.00 |
| None | 1.02 (0.66, 1.60) | 1.05 (0.67, 1.66) | 1.06 (0.67, 1.66) | 1.12 (0.73, 1.72) | 0.88 (0.54, 1.42) | 0.93 (0.59, 1.48) |
| Primary | 1.21 (0.85, 1.73) | 1.28 (0.90, 1.82) | 1.28 (0.90, 1.83) | 0.98 (0.69, 1.39) | 1.08 (0.73, 1.60) | 0.88 (0.59, 1.27) |
| Junior High | 1.14 (0.80, 1.61) | 1.16 (0.82, 1.64) | 1.16 (0.82, 1.65) | 1.17 (0.84, 1.63) | 1.00 (0.68, 1.47) | 1.01 (0.70, 1.46) |
| Senior High | 1.17 (0.81, 1.70) | 1.14 (0.79, 1.66) | 1.14 (0.79, 1.66) | 1.18 (0.82, 1.71) | 1.11 (0.76, 1.64) | 1.17 (0.80, 1.70) |
| **Occupation** |  |  |  |  |  |  |
| Office worker | 1.00 | 1.00 | 1.00 | 1.00 | 1.00 | 1.00 |
| Hairdresser/Seamstress | 1.24 (0.86, 1.80) | 1.23 (0.85, 1.78) | 1.23 (0.85, 1.78) | 1.00 (0.70, 1.42) | 1.12 (0.76, 1.65) | 0.94 (0.64, 1.37) |
| Petty trader/Fish monger | 1.19 (0.85, 1.69) | 1.23 (0.87, 1.74) | 1.23 (0.87, 1.75) | 1.23 (0.87, 1.73) | 1.06 (0.72, 1.55) | 1.12 (0.76, 1.64) |
| Student | 1.15 (0.74, 1.77) | 1.15 (0.74, 1.81) | 1.16 (0.74, 1.81) | 1.38 (0.90, 2.13) | 1.11 (0.70, 1.75) | 1.35 (0.86, 2.10) |
| Housewife/Unemployed | 1.10 (0.72, 1.66) | 1.12 (0.74, 1.69) | 1.12 (0.74, 1.70) | 0.94 (0.62, 1.41) | 1.00 (0.65, 1.54) | 0.88 (0.57, 1.35) |
| **Income** |  |  |  |  |  |  |
| <GH¢100 | 1.79 (1.28, 2.50) | 1.83 (1.31, 2.56) | 1.83 (1.31, 2.57) | 1.69 (1.17, 2.42) | 1.65 (1.16, 2.34) | 1.58 (1.09, 2.29) |
| GH¢100-500 | 1.31 (0.88, 1.94) | 1.31 (0.88, 1.95) | 1.31 (0.88, 1.96) | 1.64 (1.08, 2.47) | 1.27 (0.85, 1.90) | 1.63 (1.07, 2.48) |
| > GH¢500 | 1.00 | 1.00 | 1.00 | 1.00 | 1.00 | 1.00 |

CI indicates confidence interval. GH¢ indicates Ghana cedis. RR indicates risk ratio.

Mediation fractions (%).

Income of <GH¢100: Pre-pregnancy BMI (16.9), Cooking fuel (21.7), Joint (30.1).
